# Management of pharmaceutical services in the Brazilian primary health care

**DOI:** 10.11606/S1518-8787.2017051007063

**Published:** 2017-09-22

**Authors:** Letícia Farias Gerlack, Margô Gomes de Oliveira Karnikowski, Camila Alves Areda, Dayani Galato, Aline Gomes de Oliveira, Juliana Álvares, Silvana Nair Leite, Ediná Alves Costa, Ione Aquemi Guibu, Orlando Mario Soeiro, Karen Sarmento Costa, Augusto Afonso Guerra, Francisco de Assis Acurcio

**Affiliations:** I Programa de Pós-Graduação em Ciências e Tecnologias em Saúde. Faculdade de Ceilândia. Universidade de Brasília. Brasília, DF, Brasil; II Curso de Farmácia. Faculdade de Ceilândia. Universidade de Brasília. Brasília, DF, Brasil; III Departamento de Farmácia Social. Faculdade de Farmácia. Universidade Federal de Minas Gerais. Belo Horizonte, MG, Brasil; IV Departamento de Ciências Farmacêuticas. Universidade Federal de Santa Catarina. Florianópolis, SC, Brasil; V Instituto de Saúde Coletiva. Universidade Federal da Bahia. Salvador, BA, Brasil; VI Faculdade de Ciências Médicas. Santa Casa de São Paulo. São Paulo, SP, Brasil; VII Faculdade de Ciências Farmacêuticas. Pontifícia Universidade Católica de Campinas. Campinas, SP, Brasil; VIII Núcleo de Estudos de Políticas Públicas. Universidade Estadual de Campinas. Campinas, SP, Brasil; IX Programa de Pós-Graduação em Saúde Coletiva. Departamento de Saúde Coletiva. Faculdade de Ciências Médicas. Universidade Estadual de Campinas. Campinas, SP, Brasil; X Programa de Pós-Graduação em Epidemiologia. Faculdade de Medicina. Universidade Federal do Rio Grande do Sul. Porto Alegre, RS, Brasil

**Keywords:** Pharmaceutical Services, organization & administration, Primary Health Care, Health Management, Health Services Research, National Drug Policy, Unified Health System, Assistência Farmacêutica, organização & administração, Atenção Primária à Saúde, Gestão em Saúde, Pesquisa sobre Serviços de Saúde, Política Nacional de Medicamentos, Sistema Único de Saúde

## Abstract

**OBJECTIVE:**

To identify limiting factors in the management of pharmaceutical services in the primary health care provided by the Brazilian Unified Health System (SUS).

**METHODS:**

This study was based on the data from the *Pesquisa Nacional sobre Acesso, Utilização e Promoção do Uso Racional de Medicamentos no Brasil* (PNAUM – National Survey on Access, Use and Promotion of Rational Use of Medicines), and it was conducted by interviews with professionals responsible for pharmaceutical services in Brazilian cities, in 2015. To identify the management limiting factors, we considered the organizational, operational, and sustainability indicators of the management. For the analyses, we included the weights and structure of analysis plan for complex samples. The results were expressed by frequencies and measures of central tendency with 95% confidence interval, considering the Brazilian geographic regions.

**RESULTS:**

We identified the following limiting factors: lack of pharmaceutical services in the Municipal Health Secretariat organization chart (24%) and in the health plan (18%); lack of participation of managers in the Health Board and the absence of reference to this topic in the agenda of meetings (58.4%); lack of financial autonomy (61.5%) and lack of knowledge on the available values (81.7%); lack of adoption of operational procedures (about 50%) for selection, scheduling, and acquisition; and the fact that most professionals evaluate the organization of pharmaceutical services as good and great (58.8%), despite the worrisome indicators.

**CONCLUSIONS:**

Pharmaceutical services management is currently supported by a legal and political framework that should guide and contribute to improve the pharmaceutical services in the Brazilian Unified Health System primary health care. However, there is a mismatch between the goals established by these guidelines and what is actually happening.

## RESUMO

**OBJETIVO:**

Identificar fatores condicionantes da gestão da assistência farmacêutica na atenção primária no âmbito do Sistema Único de Saúde.

**MÉTODOS:**

Estudo com dados obtidos da Pesquisa Nacional sobre Acesso, Utilização e Promoção do Uso Racional de Medicamentos no Brasil (PNAUM) – Serviços, realizada por meio de entrevistas com responsáveis pela assistência farmacêutica em municípios, em 2015. Para identificar os fatores condicionantes da gestão foram considerados indicadores organizacionais, operacionais e de sustentabilidade da gestão. Para as análises consideraram-se os pesos amostrais e a estrutura do plano de análise para amostras complexas. Os resultados foram expressos por meio de frequências e medidas de tendência central com intervalo de confiança de 95%, por regiões geográficas do Brasil.

**RESULTADOS:**

Foram identificados os fatores condicionantes: a ausência da assistência farmacêutica no organograma da secretaria (24%) e no plano de saúde (18%), a não participação dos gestores no conselho de saúde e a não referência desse tema na pauta das reuniões (58,4%), falta de autonomia financeira (61,5%) e conhecimento dos valores disponíveis (81,7%), falta de adoção de procedimentos operacionais em cerca de 50% para seleção, programação e aquisição e o fato da maioria avaliar a organização da assistência farmacêutica como boa e ótima (58,8%), apesar dos indicadores preocupantes apontados.

**CONCLUSÕES:**

A gestão da assistência farmacêutica encontra-se respaldada em um arcabouço legal e político, que deveria nortear e contribuir para melhoria da assistência farmacêutica na atenção primária no Sistema Único de Saúde. No entanto, há um descompasso entre os objetivos fixados por essas normativas e o que se observa na realidade.

## INTRODUCTION

Implementing pharmaceutical services (PS) is pointed out as one of the challenges for the consolidation of the Brazilian Unified Health System (SUS)^8^. One of the aspects that contribute to deal with this issue is directly related to the development of its management within SUS – assumed to ensure the accessibility to medicines and integrality of therapeutic assistance^21^. However, Brazilian cities have several weaknesses regarding the decentralization of activities and PS management capacity, especially in primary health care^8^ ^,^ ^12^.

In Brazil, the concern with access to medicines and PS decentralization was improved only ten years after the creation of SUS, with the *Política Nacional de Medicamentos* (PNM – National Drug Policy) and the *Política Nacional de Assistência Farmacêutica* (PNAF – National Policy of Pharmaceutical Services)^19^. Since then, the Brazilian Ministry of Health has been providing new guidelines for pharmaceutical services to avoid restricting logistics actions, and thus improve the management and quality of these services^21^. Despite the advances, the constant problems in managing PS experienced by Brazilian cities show the difficulties still faced by SUS – regarding the quality of the pharmaceutical services provided – to ensure access and the effectiveness of health actions^18^.

Some authors point out a gap between the legislation and management practices in the PS organization in primary health care and the reality faced by Brazilian cities^2^ ^,^ ^12^. It is easy to conclude that many efforts are still needed to enhance the operational capability of these cities in the PS decentralization process^3^, mainly the need to strengthen it so that the PNM goals can be put into practice^21^.

In this sense, the development of actions to evaluate the PS management in SUS is essential to support the decision-making process and its qualification. A recent study^3^ with cities in Bahia used indicators of organizational, operational, and sustainability dimensions. These indicators were considered adequate to assess the PS management, since they contemplate logistic cycle actions and may be able to represent strategic and sustainable actions of the management results^3^.

The *Pesquisa Nacional sobre Acesso, Utilização e Promoção do Uso Racional de Medicamentos no Brasil – Serviços* (PNAUM – National Survey on Access, Use and Promotion of Rational Use of Medicines in Brazil– Services) aimed to characterize the organization of pharmaceutical services in primary health care of SUS, to promote access and the rational use of medicines, as well as to identify and discuss the factors that affect the pharmaceutical services consolidation in the municipal context.

This article is part of PNAUM – Services and aimed to identify limiting factors for PS management in primary health care of SUS, from the perspective of their managers in the five Brazilian regions.

## METHODS

PNAUM is a cross-sectional, exploratory, evaluative study, consisting of a survey with a representative sample of primary health care services in the Brazilian cities. Several study populations were considered in the sampling, with samples stratified by Brazilian regions, which are the study domains^1^. In-person interviews were held with patients, doctors, and those responsible for delivering medicines in primary health care services of SUS, in addition to observation of the pharmaceutical services facilities and telephone interviews with the professionals responsible for pharmaceutical services in the cities. Data were collected between July and December 2014.

In this article, we used data collected from interviews with the managers responsible for PS in the 600 cities from the PNAUM sample. The interviews were conducted with a structured questionnaire that had blocks of questions regarding: interviewee’s profile; PS structure and organization; PS cycle activities; social control; information and monitoring of the use of medicines. The PNAUM – Services methodology, as well as the sampling process, are described in detail by Álvares et al.^1^ (2016).

The identification of the PS management limiting factors occurred in three stages. In stage 1, we selected the indicators from the questionnaires of PS managers, having as a basis the blocks of organizational, operational, and sustainability indicators used for the same purpose by Barreto and Guimarães^3^ (2010). In stage 2, we performed the critical analysis of the results generated by the indicators. Finally, in stage 3, the indicators were categorized into blocks according to the types of limiting factors observed by the authors.

Management is a technical, political, and social process capable of producing results^3^. It is institutionalized by the formalization of structures, processes, routines, and procedures in a complex environment characterized by several factors related to the problem in question^17^.

We analyzed the data with SPSS® Statistics, version 21. For the analyses, we included the weights and structure of the analysis plan for complex samples. The results were expressed by frequencies for categorical variables and measures of central tendency for numerical variables – by Brazilian geographic regions –, both with 95% confidence interval (95%CI).

To evaluate the difference between ratios, we adopted the Chi-square test. The assessment of the differences between means was performed with generalized linear models, and the Bonferroni test was adopted for multiple comparisons. The analyses with p ≤ 0.05 were significant.

All participants signed an informed consent form. PNAUM was approved by the National Research Ethics Committee of the National Health Council under opinion no. 398,131/2013.

## RESULTS

From the 600 cities of the PNAUM – Services sample, we used data from interviews with 506 PS managers in the cities. Most respondents were women (62%), with average age of 34.8 years; 53,7% civil-service certified, and average duration of time in the post of 40 months.

Among the indicators, we identified the following limiting factors for the PS management in primary health care of SUS: PS as organizational element (factor 1); participatory management (factor 2); technical/managerial capacity (factor 3); monitoring technology and evaluation (factor 4); and management result (factor 5).

In Table 1, we present the indicators for limiting factor 1. We verified that, in about 20% of the cities studied in the Brazilian regions, pharmaceutical services were not included in the Municipal Health Secretariat’s agenda.

**Table 1:** Indicators of limiting factor 1 (pharmaceutical services as organizational element), in the primary health care of SUS, by region of Brazil. National Survey on Access, Use and Promotion of Rational Use of Medicines – Services, 2015.

Indicator	North	Northeast	Midwest	Southeast	South	Brazil	p
					
	n (%) (95%CI)	n (%) (95%CI)	n (%) (95%CI)	n (%) (95%CI)	n (%) (95%CI)	n (%) (95%CI)	
PS in the organizational chart of the Municipal Health Secretariat/FD (n = 504)	62 (63.8) (53.3–73.1)	76 (78.3) (67.9–86.1)	76 (75.1) (65.4–82.7)	84 (80.0) (70.7–86.9)	78 (72.0) (62.5–79.9)	376 (76.0) (71.4–80.1)	0.343
PS in the City/District Health Plan (n = 504)	72 (74.2) (64.1–82.2)	88 (92.3) (84.0–96.5)	77 (76.1) (66.5–83.6)	81 (74.9) (65.2–82.6)	91 (83.1) (74.5–89.2)	409 (82.0) (77.9–85.5)	0.001
Cities/FD with PTC (n = 503)	14 (10.7) (6.0–18.3)	16 (9.5) (4.8–18.0)	16 (13.6) (8.2–21.9)	19 (13.3) (7.8–21.7)	23 (15.6) (9.8–23.9)	88 (12.5) (9.5–16.3)	0.628
Meeting of the PTC with minimum regularity every six months in Cities/FD (n = 105)	8 (40.4) (19.6–65.3)	13 (62.4) (30.7–86.2)	8 (38.9) (18.7–63.9)	13 (57.3) (32.4–79.0)	15 (43.8) (24.5–65.2)	57 (51.5) (38.8–63.9)	0.934
Cities/FD that have list of medicines (n = 495)	69 (70.6) (60.3–79.1)	85 (90.8) (81.8–95.6)	81 (82.9) (73.7–89.2)	89 (86.9) (78.3–92.4)	91 (82.0) (73.2–88.4)	415 (85.3) (81.4–88.5)	0.077
Cities/FD with update of the list of medicines at least annually (n = 419)	56 (82.9) (71.8–90.3)	69 (83.8) (73.3–90.7)	65 (83.1) (73.5–89.7)	72 (81.2) (71.1–88.3)	66 (73.1) (62.6–81.5)	328 (80.4) (75.6–84.5)	0.333
Cities with permanent commission of exclusive bidding for the acquisition of medicines	34 (37.2) (27.9–47.7)	26 (30.1) (20.9–41.3)	43 (44.8) (35.1–55.0)	39 (35.7) (26.6–45.9)	40 (35.0) (26.4–44.8)	182 (34.8) (30.0–39.9)	0.574

PS: Pharmaceutical Services; FD: Federal District; PTC: Pharmacy and Therapeutics Committee.

Source: PNAUM Services – Brazil, 2015.

A significant difference was found between the regions regarding the indicator of PS as part of the city/district health plan – PS were not included in the health plans of 18% of the cities. Most respondents (85.3%) said their cities had a standard list of medicines. However, only 12.5% declared the existence of a Pharmacy and Therapeutics Committee (PTC) recognized by decree – and only 50% of the committees had meetings more than once in a semester. Another important aspect was that less than half (34.8%) of the Brazilian cities had specific bidding commissions for the purchase of medicines.

Regarding limiting factor 2, the indicators showed that less than half of Brazilian cities (about 30%) had mechanisms for patients and workers to complain and make suggestions for PS. Almost 60% of respondents answered that the managers took part in *Conselhos Municipais de Saúde* (CMS – Municipal Health Councils). However, 39.1% reported that PS were not part of the topics discussed in the meetings (Table 2).

**Table 2:** Indicators of limiting factor 2 (participatory management of pharmaceutical services), in the primary health care of SUS, by region of Brazil. National Survey on Access, Use and Promotion of Rational Use of Medicines – Services, 2015.

Indicator	North	Northeast	Midwest	Southeast	South	Brazil	p
					
	n (%) (95%CI)	n (%) (95%CI)	n (%) (95%CI)	n (%) (95%CI)	n (%) (95%CI)	n (%) (95%CI)	
Cities with accountability of the expenses with PS made on the CMS of the city (n = 323)	38 (56.0) (43.6–67.7)	32 (63.9) (48.7–76.8)	41 (63.6) (50.6–74.3)	40 (61.1) (48.5–72.4)	46 (65.4) (53.0–76.0)	197 (62.6) (56.1–68.7)	0.670
Cities where the manager of PS takes part in the CMS (n = 410)	39 (44.5) (34.2–55.4)	25 (38.1) (26.4–51.5)	41 (51.2) (39.9–62.3)	37 (41.9) (31.7–52.9)	36 (40.6) (30.5–51.5)	178 (41.6) (36.1–47.4)	0.854
Cities where the CMS decides on issues concerning PS (n = 420)	51 (58.2) (47.2–68.4)	43 (59.3) (46.5–71.0)	58 (70.2) (59.1–79.4)	54 (60.2) (49.3–70.1)	55 (61.5) (50.7–71.3)	261 (60.9) (55.2–66.3)	0.632
Cities that have mechanisms to receive criticism and suggestions from users about PS (n = 506)	35 (38.8) (29.2–49.3)	26 (24.3) (16.1–34.8)	34 (33.0) (24.3–42.9)	38 (35.8) (26.8–45.9)	43 (37.4) (28.5–47.2)	176 (32.7) (28.1–37.7)	0.566
Cities that have mechanisms to receive criticisms and suggestions from the health workers about PS (n = 420)	25 (28.8) (20.1–39.6)	18 (23.8) (14.7–36.2)	34 (41.1) (30.7–52.3)	33 (35.5) (25.9–46.4)	34 (36.5) (26.9–47.3)	144 (32.4) (27.4–37.9)	0.434

PS: Pharmaceutical Services; CMS: Municipal Health Council.

Source: PNAUM Services – Brazil, 2015.

Table 3 shows the indicators that characterize limiting factor 3. Among them, we observed that the percentage of cities that invested in the PS structuring process was less than 50%. Of the 499 respondents, 220 (44.3%) said that their cities – or Brazilian Federal District – invested in the structuring of the services (repairs, construction, among others) and, among them, 66.5% received no transfers from the states and from the Brazilian Government for this purpose. Most managers (81.7%) did not know how much was spent with PS in the city or in the Federal District. More than half (61.5%) of these managers said they have no management autonomy for PS financial resources.

**Table 3:** Indicators of limiting factor 3 (technical/managerial capacity of the management of pharmaceutical services), in the primary health care of SUS, by region of Brazil. National Survey on Access, Use and Promotion of Rational Use of Medicines – Services, 2015.

Indicators	North	Northeast	Midwest	Southeast	South	Brazil	p
					
	n (%) (95%CI)	n (%) (95%CI)	n (%) (95%CI)	n (%) (95%CI)	n (%) (95%CI)	n (%) (95%CI)	
Educational level of the manager (n = 504)							0.015
Up to high school	6 (6.8) (3.1–14.4)	2 (2.6) (0.6–9.8)	6 (6.2) (2.8–13.3)	3 (3.1) (1.0–9.3)	4 (4.0) (1.5–10.1)	21 (3.7) (2.2–6.1)	
Higher education degree	78 (83.3) (74.1–9.7)	66 (71.3) (60.4–80.3)	72 (74.0) (64.4–81.8)	83 (80.9) (71.8–87.6)	68 (60.3) (50.5–69.4)	367 (72.9) (68.2–77.2)	
Specialization	11 (9.9) (5.3–17.9)	26 (36.1) (17.5–36.9)	22 (19.7) (13.0–28.8)	19 (16.0) (9.9–24.7)	38 (35.7) (27.0–45.6)	116 (23.4) (19.3–28.0)	
% of cities that offer qualification or training for professionals of PS (n = 418)	9 (10.1) (5.2–18.8)	8 (11.6) (5.6–22.4)	11 (14.6) (8.3–24.6)	11 (13.3) (7.5–22.6)	11 (9.2) (4.7–17.4)	50 (11.7) (8.5–16.0)	0.800
Cities with SOP for: (n = 491)							
Selection of medicines	42 (45.9) (35.6–56.6)	46 (48.8) (37.7–60.0)	50 (51.5) (41.4–61.4)	60 (56.5) (46.3–66.1)	52 (45.9) (36.4–55.7)	250 (50.6) (45.3–55.8)	0.564
Scheduling	40 (45.0) (34.6–55.8)	50 (56.1) (44.7–67.0)	53 (53.0) (43.0–62.9)	68 (64.6) (54.4–73.7)	48 (43.2) (33.8–53.1)	259 (54.7) (49.5–59.9)	0.038
Acquisition	47 (52.6) (41.9–63.1)	52 (58.8) (47.3–69.4)	52 (52.8) (42.7–62.6)	69 (66.7) (56.5–75.5)	55 (49.4) (39.7–59.1)	275 (58.2) (52.9–63.2)	0.078
Cities where the pharmacist makes the technical specification of medicines to be purchased (n = 505)	77 (80.4) (70.9–87.4)	78 (83.1) (73.1–89.9)	90 (90.4) (82.7–94.9)	91 (89.3) (81.4–94.1)	96 (88.0) (80.1–93.0)	432 (86.6) (82.6–89.7)	0.352
Cities where there is purchase of medicines in local pharmacies (n = 499)							< 0.001
Always	4 (4.5) (1.7–11.4)	2 (2.6) (0.6–9.9)	16 (17.0) (10.6–26.0)	11 (11.7) (6.6–20.1)	12 (12.0) (6.9–20.0)	45 (9.0) (6.5–12.3)	
Repeatedly	4 (4.5) (1.7–11.4)	4 (4.0) (1.3–11.5)	7 (7.4) (3.6–14.8)	4 (4.3) (1.6–10.9)	13 (13.0) (7.7–21.1)	32 (6.5) (4.4–9.4)	
Sometimes	14 (15.9) (9.5–24.8)	19 (21.0) (13.3–31.6)	35 (36.3) (27.3–46.5)	31 (31.1) (22.5–41.2)	35 (32.2) (23.8–42.0)	134 (27.6) (23.3–32.5)	
Rarely	25 (27.2) (18.9–37.3)	15 (14.6) (8.3–24.3)	17 (17.2) (10.9–26.3)	22 (20.5) (13.5–29.9)	19 (18.1) (11.7–26.9)	98 (18.4) (14.8–22.8)	
Never	41 (40.9) (31.2–51.3)	43 (46.1) (35.3–57.2)	20 (18.8) (12.2–27.9)	30 (28.0) (19.9–38.0)	26 (20.8) (14.0–29.8)	160 (31.9) (27.2–36.9)	
Cities where the ratio of the average percentage of expired medicines by the total number of acquisitions is up to 5% (n = 489)	77 (84.1) (75.1–90.3)	66 (73.9) (62.8–82.7)	80 (83.3) (74.5–89.5)	86 (85.5) (76.5–91.4)	91 (83.0) (74.3–89.2)	400 (81.3) (76.7–85.1)	0.472
Cities where the Secretariat has legal advice to answer lawsuits (n = 322)	37 (83.2) (67.9–92.1)	49 (84.8) (70.2–93.0)	62 (85.3) (74.8–92.0)	61 (80.4) (69.0–88.3)	61 (80.5) (69.2–88.3)	270 (82.1) (76.5–86.7)	0.855
Cities where the coordination of PS has autonomy for managing the financial resources destined to PS (n = 504)	53 (57.0) (46.6–66.9)	39 (43.4) (32.9–54.5)	44 (44.9) (35.3–55.0)	35 (32.7) (24.0–42.7)	36 (31.7) (23.3–41.4)	207 (38.5) (33.6–43.6)	0.079
Cities that received resources from the State or Federal Government for structuring PS in the primary health care in the past year (reforms, construction, among others) (n = 499)	23 (23.3) (15.7–33.1)	37 (44.3) (33.5–55.7)	23 (23.6) (16.1–33.2)	35 (34.6) (25.8–44.7)	27 (25.8) (18.2–35.2)	145 (33.5) (28.7–38.7)	0.066
Cities that spent resources in structuring PS in the past year (n = 501)	35 (34.2) (25.2–44.5)	50 (54.5) (43.3–65.1)	44 (45.9) (36.1–56.0)	35 (32.4) (23.7–42.5)	56 (50.2) (40.6–59.9)	220 (44.3) (39.3–49.5)	0.015
Managers who did not know which was the total expenditure of the city with PS in the past year (n = 501)	77 (85.2) (76.0–91.2)	67 (76.8) (65.9–85.0)	84 (88.5) (80.5–93.5)	79 (85.6) (77.0–91.7)	82 (79.0) (69.8–86.0)	389 (81.7) (77.2–85.5)	0.213
Managers who consider that the list of medicines adopted by the city meets the population demands (n = 501)							0.349
Completely	16 (24.6) (15.6–36.5)	24 (27.2) (18.1–38.7)	25 (29.3) (20.4–40.1)	32 (34.6) (25.1–45.5)	27 (27.8) (19.2–38.2)	124 (29.7) (24.7–35.1)	
Partially	55 (75.4) (63.5–84.4)	63 (72.8) (61.3–81.9)	59 (70.7) (59.9–79.6)	59 (65.4) (54.5–74.9)	65 (71.1) (60.5–79.7)	301 (70.1) (64.6–75.0)	
Evaluation of PS organization in the city/FD by the manager (n = 420)							0.852
Great	8 (9.8) (5.0–18.5)	4 (5.0) (1.6–14.3)	7 (9.3) (4.5–18.3)	7 (8.4) (4.0–16.6)	6 (7.1) (3.2–15.1)	32 (7.4) (4.9–10.9)	
Good	38 (42.0) (31.9–52.9)	39 (56.4) (43.8–68.3)	42 (54.8) (43.5–65.6)	44 (48.2) (37.7–58.9)	48 (52.1) (41.4–62.5)	211 (51.4) (45.7–57.0)	
Regular	32 (38.3) (28.4–49.3)	22 (30.0) (19.8–42.6)	25 (29.2) (20.1–40.3)	32 (36.1) (26.5–46.9)	25 (28.7) (20.0–39.3)	136 (32.3) (27.3–37.9)	

PS: Pharmaceutical Services; FD: Federal District; SOP: Standard Operating Procedure.

Source: PNAUM Services – Brazil, 2015.

No standard operating procedures (SOP) for the selection of medicines was reported in approximately 50% of the cities, and, in 13.4% of the cities (or Federal District), pharmacists were not the professionals responsible for the technical specifications in the purchase of medicines – even though more than 90% of PS managers were pharmacists. About 80% of the cities had a ratio of expired medicines higher than 5% in relation to the total of purchases.

We also observed a high percentage of cities (82.1%) where the Municipal Health Secretariat relied on legal advice to deal with lawsuits. Although most managers (80.4%) said the list of medicines adopted by the city was updated at least once a year, 70.1% considered that the SMS met population demands only partially. Most of them (58.8%) considered the PS organization “good” and “great.”

Concerning limiting factor 4, almost 30% of respondents reported that the city had no computerized system for PS management; less than half was connected with other health units; and only 34.2% do not use it for the control and use of funds.

About 80% of the managers reported that no mechanisms were available for the registration of technical complaints and notification of adverse events. Concerning the conditions of the storage of medicines, the items “humidity” and “temperature” stand out with lower percentages of monitoring (Table 4).

**Table 4:** Indicators of limiting factor 4 (monitoring technology and evaluation of the management of pharmaceutical services), in the primary health care of SUS, by region of Brazil. National Survey on Access, Use and Promotion of Rational Use of Medicines – Services, 2015.

Indicators	North	Northeast	Midwest	Southeast	South	Brazil	p
					
	n (%) (95%CI)	n (%) (95%CI)	n (%) (95%CI)	n (%) (95%CI)	n (%) (95%CI)	n (%) (95%CI)	
Cities with some mechanism for the registration of technical complaint and notification of adverse events of medicines (n = 417)	19 (22.2) (14.5–32.5)	15 (15.9) (8.6–27.5)	19 (24.6) (16.1–35.6)	21 (22.9) (15.1–33.2)	23 (23.1) (15.3–33.2)	97 (21.1) (16.9–26.1)	0.212
Cities with records of expired medicines (n = 497)	77 (79.6) (69.9–86.7)	72 (75.9) (64.9–84.3)	84 (85.9) (77.4–91.6)	85 (81.7) (72.4–88.3)	94 (86.0) (77.8–91.5)	412 (81.2) (76.7–85.0)	0.119
Cities with computerized system for PS management (n = 501)	41 (40.1) (30.5–50.5)	60 (61.2) (50.0–71.4)	54 (53.5) (43.5–63.3)	81 (78.7) (69.2–85.8)	95 (87.9) (79.9–93.0)	331 (70.6) (65.9–74.9)	< 0.001
Cities with monitoring of the medicine storage conditions (n = 497)							
Temperature	71 (72.0) (61.8–80.3)	82 (88.0) (78.4–93.7)	81 (82.0) (72.8–88.5)	82 (79.4) (69.9–86.5)	94 (85.1) (76.7–90.8)	410 (82.9) (78.6–86.4)	0.101
Moisture	58 (57.4) (47.0–67.2)	73 (77.2) (66.4–85.4)	72 (72.4) (62.5–80.5)	68 (65.3) (55.1–74.3)	75 (66.3) (56.6–74.9)	346 (69.0) (64.0–73.5)	0.229
Cleaning	84 (86.6) (77.7–92.2)	85 (93.3) (84.8–97.2)	90 (92.3) (84.9–96.2)	97 (94.6) (87.6–97.8)	103 (94.0) (87.3–97.3)	459 (93.2) (90.2–95.4)	0.363
Protection from insects	74 (76.2) (66.3–83.9)	80 (87.8) (78.3–93.5)	83 (84.9) (76.1–90.8)	88 (84.9) (76.0–90.9)	97 (88.1) (80.1–93.1)	422 (85.8) (81.8–89.0)	0.183
Cities in which the computerized system includes the items: (n = 336)							
Acquisition	32 (79.0) (63.0–89.3)	48 (79.1) (65.5–88.3)	46 (84.3) (71.6–92.0)	73 (87.1) (77.7–92.9)	79 (84.0) (74.8–90.3)	278 (83.7) (78.6–87.8)	0.837
Financial control and execution	27 (69.2) (52.9–81.8)	39 (69.8) (55.5–81.1)	36 (67.7) (53.8–79.0)	53 (64.0) (52.8–73.8)	60 (63.6) (53.2–73.0)	215 (65.8) (59.7–71.4)	0.723
Inventory storage and control	35 (87.4) (72.7–94.8)	55 (91.7) (79.9–96.8)	54 (100) (100–100)	75 (88.4) (79.2–93.9)	85 (88.6) (80.1–93.8)	304 (90.0) (85.5–93.2)	
Delivery of medicines to the user	29 (70.6) (54.1–83.1)	48 (77.2) (63.4–86.9)	48 (89.7) (78.1–95.5)	80 (94.8) (87.1–98.0)	93 (97.7) (91.3–99.4)	298 (89.8) (85.4–93.0)	0.036
Cities where the computerized system is networked with the UBS (n = 331)	14 (29.6) (17.6–45.1)	26 (33.2) (21.9–46.1)	34 (57.4) (44.0–69.8)	39 (44.3) (33.7–55.5)	59 (62.9) (52.4–72.3)	172 (46.6) (40.7–52.6)	0.009
Cities where the computerized system has user registration – Patient Record (n = 336)	8 (22.4) (11.6–38.8)	20 (37.2) (24.8–51.4)	25 (46.1) (33.0–59.7)	51 (62.6) (51.4–72.6)	57 (58.9) (48.3–68.7)	161 (52.4) (46.3–58.4)	0.004

PS: Pharmaceutical Services; UBS: Basic Health Unit.

Source: PNAUM Services – Brazil, 2015.

Finally, regarding limiting factor 5 (management result), the indicator of availability of medicines showed that 21.3% (95%CI 17.5–25.9) of Brazilian cities had periods of shortage of supply in the past year, with statistically significant difference (*p*=0.003) between regions: North (34.6%; 95%CI 25.0-45.5), Northeast (13.8%; 95%CI 7.6-23.8), Midwest (29.8%; 95%CI 21.4-39.8), Southeast (29.8%; 95%CI 21.4-39.8), South (24.7%; 95%CI 17.2-34.1).

## DISCUSSION

The indicators used in this study indicated limiting factors that still exist in PS management at national level, including the importance of PS management in its interrelation with the health system, its aspects and actors involved in the process.

PS did not appear in the organizational structure of SMSs in 25% of the cities investigated. This can affect their inclusion in the health planning process and affect the implementation of their activities. The organizational structure comprises identification, analysis, classification, and grouping of activities and resources, to reach the results previously set by the planning^16^.

Authors point out that the way how PS are organized within SUS limits their field of activity, and the fragmentation of their activities may contribute to the disruption with the other health actions^3^. Not being in the cities’ health plans is an obstacle to PS management, since their actions must be planned in the context of the other health actions and declared as commitment in the health plans^14^. Health plans are central tools of planning and they must be prepared from a situational analysis, reflecting the population’s health needs and serving as a basis for the implementation, monitoring, evaluation and practice of the management of the cities’ health system^15^.

Although 85.3% of the surveyed cities in the different regions have answered they adopt a list of medicines, the vast majority (87.5%) had no formalized PTC, and when they had, it did not meet periodically. In the management process, the PTC plays an important role related to the selection of medicines, especially essential ones, which are those that treat most diseases or the main health problems that affect a population^11^. It is worth highlighting that only 34.8% of the cities had a committee of specific bidding for PS, which may hinder even more the management scenario and impair the availability of safe, effective, and cost-effective medicines to the population.

Regarding limiting factor 2, the low participation of the PS manager in the CMS must also be subject of concern, since this impairs the contribution of this actor with the discussions on the topic and prevents patients’ demands to be met. In addition, managers report that some cities have no mechanisms for ombudsmanship of patients and workers, highlighting weaknesses in the management listening process. Participatory management should be institutionalized and understood as part of the guarantee of the right to health. Participation in health was established in the article 198 of the Brazilian Constitution, regulated by law 8,080/90^4^ and supplemented by law 8.142/90^5^ and by decree 7.508/2011^6^, which set the practice of social control by conferences and health councils, establishing a pattern of representativeness in the construction, operation, and management of social policies, especially those of health. Nevertheless, 39.1% of managers say that PS are not part of the topics discussed within the CMS, showing a gap between the National Policy of PS and the guidelines employed in the construction of SUS management.

Authors say that it is possible to distinguish a specific area of PS related to the technology of medicine management, so as to ensure the supply and access and, therefore, the demand of a specific profile for the execution of the activities^2^. Despite the high education level of those responsible for PS in Brazil – most with higher education, with 90.7% graduates and about 20% specialists in Pharmacy –, we verified that only 11.7% of the cities offer qualification or training for professionals of PS. The results indicate a degree of lack of incentive to the training of professionals with specific skills for management, considering the complexity of the topic and the characteristics of the public sector and primary health care. This is a challenge to be overcome for the qualification of management, since the legislation of this field^a^ guides a series of responsibilities for the pharmacist in the area of management.

The technology of management is also characterized by preestablished procedures and a set of activities required for the fulfillment of the legal aspects of health policies^2^. Under this perspective, several indicators that corresponded to the technical/managerial capacity of PS were also assessed in previous studies^8^ ^,^ ^18^. Although starting from different methodological approaches, many of the results described by the authors are consistent with those observed by this research such as noncompliance with standards for acquisition of medicines, lack of financial contribution, and expired medicines.

The percentage of cities with noncompliance with standards for acquisition of medicines described in a research carried out in cities of the state of Paraíba^8^ was 52.7%, which was similar to our findings. However, a study carried out from auditing reports of the Comptroller General in Brazilian cities^18^ presented lower results (19.4%). The frequency of cities with lack of financial contribution found in these studies was 20%^18^ and 13.6%^8^, and the existence of expired medicines was 13.2%^18^ and 10.9%^8^. In our findings, the frequencies were a little higher for these indicators: 21.3% and 33.5%, respectively.

About 50% of the sample reported lack of SOP for selection, scheduling, and acquisition of medicines. The lack or deficiency in these processes may trigger successive errors that will result in losses and waste of public resources, affecting the access to medicines and hindering the guarantee of a comprehensive health care, which is one of the SUS principles^18^. In 13.4% of the cities, it is not the pharmacist who makes the technical specifications for the purchase of medicines, and, in almost 30% of the cities, we observed the purchase of medicines in local pharmacies. This can reveal the dimension of the problem of management in these processes, since the bidding process has as one of its principles selecting the most advantageous proposal for the public administration^7^. Public audits showed that the acquisition took place without the bidding process^8^ in 14.5% of the cities in the Northeast.

The manager’s autonomy on financial resources is essential for the decision-making process^3^. In this sense, the lack of financial autonomy declared by 61.5% of professionals responsible for PS, associated with the fact that 81.7% do not know how much was spent by the city with PS, may determine significant limitations in performing the management effectively. In the case of the transfer of funds from other areas, less than 50% of cities invested its own resources in structuring PS, raising the following possibilities: PS are already structured; structuring is not yet a concern of the management; or there are no resources. A small percentage of cities received State and federal support. The funding of PS must be agreed between the Federal Government, States, Federal District, and cities. However, since one admits an underfunding of SUS, one also expects problems in the management of PS^20^.Despite a series of difficulties that still exist in the reality of decentralized PS, most (58.8%) managers evaluated the organization as good and great.

The use of a computerized system to support the management activities of PS has been encouraged by the Brazilian Ministry of Health^10^, but the indicators of limiting factor 4 pointed to some obstacles. Almost 30% of cities reported not having a computerized system for the management of PS. Besides, although the system provided to SUS being free and having possibilities for managing a series of information and sharing with the health network, 34.2% of the sampled cities did not use it for control and use of funds, and fewer than half of the systems were networked.

To show the forms of monitoring and evaluation of the management, we considered the use of mechanism for the registration of technical complaints and notification of adverse events, as well as the registration of expired medicines and control of storage conditions. These two items must be systematic, continuous, and provide information that allows a quick situational evaluation. The absence of mechanisms to help problem identification and decision-making will affect the management^9^. In view of the high percentage of lack of mechanisms for registration of technical complaints and notification of adverse events in the cities (79.9%), the need to invest in their creation is clear, since they are essential instruments for pharmacovigilance activities, fulfilling one precept of PNM.

Although PNAF shifts the focus of PS actions from the medicine to the user, the medicine must be available so that the recommended access with rational use can occur. And, as the management seeks to achieve objectives in line with its context^17^, one of the purposes of PS management certainly is to meet the precepts of the existing legislation^13^ and ensure the access of the population to medicines. Under this light, the shortage was perceived as an indicator for limiting factor 5, in which about 20% of cities in Brazil claimed to have had a period of shortage in the past year. We observed significant difference (p=0.003) between regions, especially in the Northeast (13.8%), with minor shortages, and in the North, with higher percentage of cities facing shortages of medicines in the past year (34.6%). A previous study^18^ described a similar frequency of shortages for the national context (24.1%). However, cities in the Northeast presented higher prevalence of shortages (30.3%)^8^. The differences can be due to improvements in the management of PS in the Northeast, or could be justified by the different methodological approaches of the research.

The critical and integrated analysis of the results of this research, by managers and other health actors participating in the process of PS management, can help facing the challenges that prevail in primary health care, in the five Brazilian regions. The management of PS is supported in a legal and political framework, which should guide and contribute to improvements in their process and effectiveness of actions. However, there is a mismatch between what is proposed by these guidelines and what is seen in reality, demonstrated by the indicators and limiting factors analyzed.

One must recognize the limiting factors of PS management, especially those critical to the quality of the actions and that hinder the goals of PS policies in SUS. These factors are likely to change, according to the situation and needs, affecting the decision-making process, the implementation of new actions, and future plans for the area. The advances in the structuring of management processes are undeniable, and they were especially driven by the law and by the guidelines of the Brazilian Ministry of Health, by incentives in the organization of PS in primary health care and by the establishment of funding for PS in the SUS. However, our results show that the gaps between the legally established PS and the PS experienced in primary health care, in the Brazilian regions, widely involve the limiting factors of their management.

## Footnotes

a Conselho Federal de Farmácia (CFF). Resolução nº 578, de 26 de julho de.2013. Ementa: Regulamenta as atribuições técnico-gerenciais do farmacêutico na gestão da assistência farmacêutica no âmbito do Sistema Único de Saúde (SUS). Brasília (DF); 2013 [cited 2017 Jan 26]. Available from: http://www.cff.org.br/userfiles/file/resolucoes/578.pdf

## References

[1] Álvares J, Alves MCGP, Escuder MML, Almeida AM, Izidoro JB, Guerra AA (2017). Pesquisa Nacional sobre Acesso, Utilização e Promoção do Uso Racional de Medicamentos: métodos. Rev Saude Publica.

[2] Araújo ALA, Ueta JM, Freitas O (2005). Assistência farmacêutica como um modelo tecnológico em atenção primária à saúde. Rev Cienc Farm. Basica Apl.

[3] Barreto JL, Guimarães MCL (2010). Avaliação da gestão descentralizada da assistênica farmacêutica básica em municípios baianos, Brasil. Cad Saude Publica.

[4] Brasil (1990). Lei nº 8.080 de 19 de setembro de 1990. Dispõe sobre as condições para a promoção, a organização e o funcionamento dos serviços correspondentes e dá outras providências. Diario Oficial Uniao.

[5] Brasil (1990). Lei nº 8.142, de 28 de dezembro de 1990. Dispõe sobre a participação da comunidade na gestão do Sistema Único de Saúde (SUS) e sobre as transferências intergovernamentais de recursos financeiros na área da saúde e dá outras providências. Diario Oficial Uniao.

[6] Brasil (2011). Decreto nº 7.508, de 28 de junho de 2011. Regulamenta a Lei nº 8.080, de 19 de setembro de 1990, para dispor sobre a organização do Sistema Único de Saúde - SUS, o planejamento da saúde, a assistência à saúde e a articulação interfederativa, e dá outras providências. Diario Oficial Uniao.

[7] Brasil (1993). Lei nº 8.666, de 21 de junho de 1993. Regulamenta o art. 37, inciso XXI, da Constituição Federal, institui normas para licitações e contratos da Administração Pública e dá outras providências. Diario Oficial Uniao.

[8] Bruns SF, Luiza VL, Oliveira EA (2014). Gestão da assistência farmacêutica em municípios do estado da Paraíba (PB): olhando a aplicação de recursos públicos. Rev Adm Publica.

[9] Carvalho ALB, Souza MF, Shimizu HE, Senra IMVB, Oliveira KC (2012). A gestão do SUS e as práticas de monitoramento e avaliação: possibilidades e desafios para a construção de uma agenda estratégica. Cienc Saude Coletiva.

[10] Costa KS, Nascimento JM (2012). HÓRUS: inovação tecnológica na assistência farmacêutica no Sistema Único de Saúde. Rev Saude Publica.

[11] Marin N, Luzia VL, Osório de Castro CGE, Machado dos Santos S (2003). Assistência farmacêutica para gerentes municipais.

[12] Mendes SJ, Manzini F, Farias MR, Leite SN (2015). Gestão da Assistência Farmacêutica: avaliação de um município catarinense. Rev Eletron Gestão Saude.

[13] Ministério da Saúde (BR), Conselho Nacional de Saúde (2004). Resolução nº 338, de 06 de maio de 2004. Aprova a Política Nacional de Assistência Farmacêutica. Diario Oficial Uniao.

[14] Ministério da Saúde (BR), Secretaria de Ciência, Tecnologia e Insumos Estratégicos, Departamento de Assistência Farmacêutica e Insumos Estratégicos (2006). Planejar é preciso: uma proposta de método para aplicação à assistência farmacêutica.

[15] Ministério da Saúde (BR) (2013). Portaria nº 2.135, de 25 de setembro de 2013. Estabelece diretrizes para o processo de planejamento no âmbito do Sistema Único de Saúde (SUS).

[16] Oliveira DPR (2006). Estrutura organizacional: uma abordagem para resultados e competitividade.

[17] Tanaka OY, Tamaki EM (2012). O papel da avaliação para a tomada de decisão na gestão de serviços de saúde. Cienc Saude Coletiva.

[18] Vieira FS (2008). Qualificação dos serviços farmacêuticos no Brasil: aspectos inconclusos da agenda do Sistema Único de Saúde. Rev Panam Salud Publica.

[19] Vieira FS (2010). Assistência farmacêutica no sitema público de saúde do Brasil. Rev Panam Salud Publica.

[20] Vieira FS, Zucchi P (2013). Financiamento da assistência farmacêutica no Sistema Único de Saúde. Saude Soc.

[21] Vieira FS, Zucchi P (2014). Gestão da assistência farmacêutica: análise da situação de alguns municípios. Tempus Actas Saude Coletiva.

